# A study of longitudinal tumor motion in helical tomotherapy using a cylindrical phantom

**DOI:** 10.1120/jacmp.v14i2.4022

**Published:** 2013-03-04

**Authors:** Michael Klein, Stewart Gaede, Slav Yartsev

**Affiliations:** ^1^ Department of Physics London Regional Cancer Program, London Health Sciences Centre; ^2^ Department of Oncology University of Western Ontario London Ontario Canada

**Keywords:** tomotherapy, tumor motion, ArcCHECK

## Abstract

Tumor motion during radiation treatment on a helical tomotherapy unit may create problems due to interplay with motion of the multileaf collimator, gantry rotation, and patient couch translation through the gantry. This study evaluated this interplay effect for typical clinical parameters using a cylindrical phantom consisting of 1386 diode detectors placed on a respiratory motion platform. All combinations of radiation field widths (1, 2.5, and 5 cm) and gantry rotation periods (16, 30, and 60 s) were considered for sinusoidal motions with a period of 4 s and amplitudes of 5, 6, 7, 8, 9, and 10 mm, as well as real patient breathing pattern. Gamma comparisons with 2% dose difference and 2 mm distance to agreement and dose profiles were used for evaluation. The required motion margins were determined for each set of parameters. The required margin size increased with decreasing field width and increasing tumor motion amplitude, but was not affected by rotation period. The plans with the smallest field width of 1 cm have required motion margins approximately equal to the amplitude of motion (±25%), while those with the largest field width of 5 cm had required motion margins approximately equal to 20% of the motion amplitude (±20%). For tumor motion amplitudes below 6 mm and field widths above 1 cm, the required additional motion margins were very small, at a maximum of 2.5 mm for sinusoidal breathing patterns and 1.2 mm for the real patient breathing pattern.

PACS numbers: 87.55.km, 87.55.Qr, 87.56.Fc

## I. INTRODUCTION

Motion of the internal organs may create problems in radiation therapy, which relies on a static target to plan a dose delivery. Breathing can result in lung tumor motion with amplitudes above 5 mm and periods close to 4 s, mainly in the superior–inferior (SI) direction.^(^ ^1^ ^–^ ^3^ ^)^ However, breathing‐induced motion is not limited to the lungs; the liver and kidney may move by 1.5–2 cm in the SI direction during normal respiration.^(^ ^4^ ^)^ Pancreatic tumors may move by almost 1 cm in the SI direction and half that in the anterior–posterior (AP) direction.^(^ ^5^ ^)^ This ‘intrafraction’ motion can cause unwanted effects, such as the blurring of the intended dose distribution (dose rounding) along the direction of motion.

The problem is further complicated in intensity‐modulated radiation therapy (IMRT), including helical tomotherapy (HT), because of the additional dynamics associated with these forms of radiation therapy. HT involves multileaf collimator (MLC) motion, rotation of the entire linear accelerator on a gantry around the target, and the translation of the patient couch through the gantry. With thoracic tumors that move due to breathing, the interplay between all of these motions may lead to artifacts in the dose distribution. These artifacts have been identified as dose rounding, dose rippling, and the leaf opening asynchronization effect.^(^ ^6^ ^)^ Some authors have suggested that either the interplay effect itself does not produce significant error beyond dose rounding for sinusoidal breathing patterns,^(^ ^7^ ^–^ ^10^ ^)^ or that the error is reduced over several fractions.^(^ ^7^ ^,^ ^11^ ^–^ ^13^ ^)^ Other studies have found that there are significant effects, even with sinusoidal motion^(^ ^6^ ^)^ or with only real patient breathing patterns.^(^ ^9^ ^,^ ^14^ ^)^

The standard method for dealing with uncertainties such as tumor motion is to expand the clinical target volume (CTV) by a certain margin to create a planning target volume (PTV). The PTV must be large enough to ensure that even with motion, the CTV would be covered by the prescribed dose. Because the required size of the margin is uncertain, physicians must overestimate the necessary PTV size in order to ensure coverage. This leads to an increase in the level of radiation given to healthy tissue.

Other methods have been proposed to combat motion‐induced dose artifacts in IMRT. Respiratory gating is a technique used in IMRT in which radiation delivery is shut off when the tumor is not within a predefined area.^(^ ^15^ ^,^ ^16^ ^)^ Unfortunately, in HT, the constant couch translation and gantry rotation do not allow for a pause in radiation delivery. Some HT‐specific solutions include several deliveries with a large pitch factor,^(^ ^17^ ^)^ rearranging sinogram projections to account for target motion,^(^ ^18^ ^)^ incorporating CT images from different breathing phases into the plan optimization process,^(^ ^19^ ^)^ and creating intensity maps with increased intensity near the edges of the tumor, in addition to margins.^(^ ^20^ ^)^ At the present time, none of these have been implemented into clinical use. Margin expansion is still used clinically and although it is an imperfect solution, it may be improved upon by optimizing margin sizes in order to completely cover the CTV while sparing as much healthy tissue as possible.

The dose distribution delivered to the moving target depends on the relation between motions of the target, gantry, couch, and MLCs. We were interested to see how different parameters of the planned delivery affect the dose distribution in a moving target. The objectives of this study are three‐fold. The effects caused by changes in various machine and target motion parameters are analyzed in an attempt to define the machine parameters which should be used in treatments involving tumor motion, to predict optimal margin sizes for various situations, and to identify differences in the effects of sinusoidal versus real patient breathing motions.

## II. MATERIALS AND METHODS

**Figure 1:**
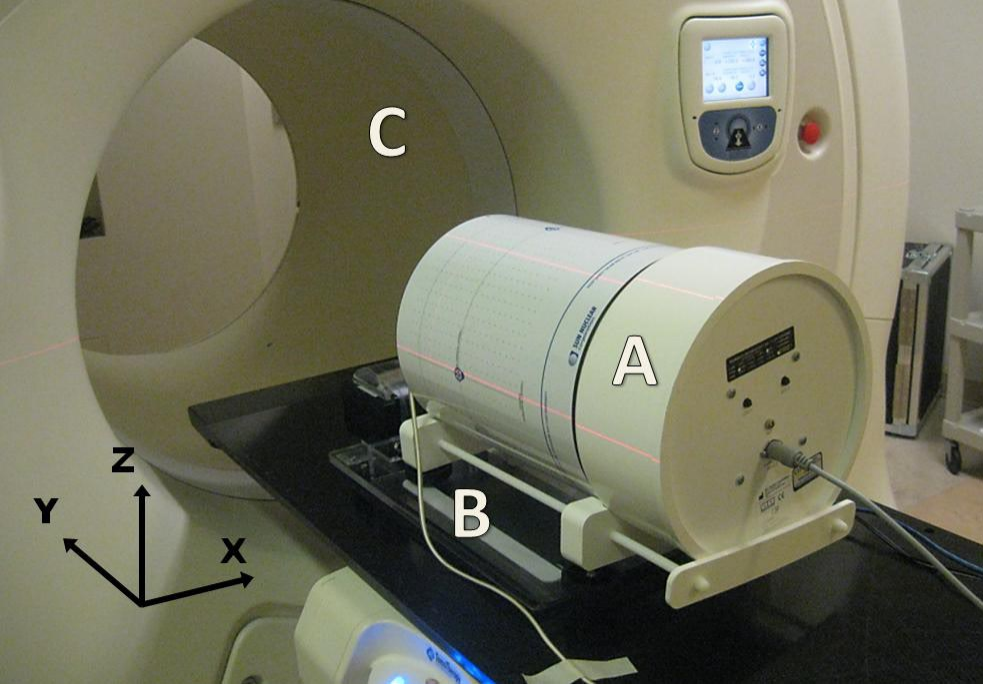
ArcCHECK phantom (A) placed on the motion platform (B) on the couch of tomotherapy Hi·Art unit (C). Motion was in superior–inferior (y) direction.

An ArcCHECK (Sun Nuclear Corp., Melbourne, FL) phantom was placed on a QUASAR respiratory motion platform (Modus Medical Devices Inc., London, ON, Canada) (Fig. 1), to simulate a patient with moving internal organs. The ArcCHECK's cylindrical geometry is designed for rotational quality assurance. It features 1386 SunPoint Diode Detectors spiraling down the length of the cylinder, which allow it to sample the dose field accurately and with 1 cm resolution between individual detectors. The QUASAR respiratory motion platform was used to move the ArcCHECK in order to simulate breathing motion.

Using a Brilliance Big Bore scanner (Philips, Eindhoven, The Netherlands), kilovoltage CT images of the ArcCHECK on the motion platform were obtained while the platform was in a static state. These images were transferred as a phantom to the tomotherapy treatment planning system (TPS) (version 3.1.5).

A patient was created in ${\text{Pinnacle}}^{3}$ version 8.0m (Philips, Fitchburg, WI), with contours of a CTV and organ at risk (OAR). The CTV was constructed as a cylinder lying along the axis of motion, with length 33 mm and diameter 37.5 mm. The cylindrical OAR, of length 21 mm and diameter 49.2 mm, was placed at 12 mm from the CTV along the SI direction towards the gantry, since the SI direction was the direction of motion. This represented a situation involving any tumor moving together with an OAR — for example, a kidney tumor moving together with a portion of the liver.

The “patient” was transferred to the tomotherapy TPS, where nine treatment plans were created. All combinations of field widths (FW) 1.0, 2.5, and 5.0 cm and rotation periods (RP) 16, 30, and 60 s were represented in the plans. Pitches ranged from 0.215 to 0.86, and modulation factors from 1.000 (actual 1.024) to 1.550 (1.552). Variations in the pitch and modulation factor values were used to obtain the plans with desired RPs for all FW options. The dose prescription was set to 4 Gy for 95% of the CTV. All the plans were optimized with the same constrains and penalties for minimum and maximum doses to the target (set as the prescription dose in our clinical practice) and with the hard constraint of the prescribed dose of 4 Gy to 95% of the target volume.

**Figure 2:**
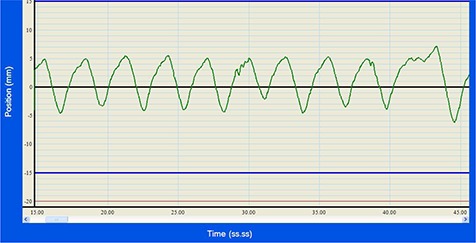
Real patient breathing pattern.

Delivery quality assurance (DQA) plans using the ArcCHECK as a phantom were created for each treatment plan. In these plans, the ArcCHECK was placed so that the diodes would pass through both the tumor and the OAR to ensure that the dose distribution would be measured in this region. The detector was placed in exactly the same position for every plan. The DQA plans were run on the tomotherapy unit with sinusoidal motion, real patient breathing motion, and no motion. The real patient breathing motion had average amplitude slightly below 5 mm and an average period of approximately 3 s (Fig. 2). The sinusoidal motions all had a period of 4 s and had amplitudes of 5, 6, 7, 8, 9, and 10 mm. For the plan with FW=5.0 cm and RP=60 s (henceforth represented as 5.0/60), two measurements were done for each set of parameters. For all other plans, three measurements were performed, for a total of 208 measurements. The three measurements were averaged together to simulate a three‐fraction treatment. In clinical practice, the delay between pressing the “start” button and the actually delivery is not fixed, because it depends on the number of gantry rotations required for the system to ensure correct gantry position and speed. As it is impossible to synchronize the phases for phantom motion and gantry; averaging over deliveries of three fractions simulates the effect of multifractional delivery in the presence of target motion.

The results were analyzed in two ways. First, gamma comparisons with 2% dose difference and 2 mm distance‐to‐agreement of individual motion measurements versus corresponding static measurements were performed using MapCHECK software (version 5.02). The measured data for three consecutive measurements representing multifractional delivery were compared to static measurements, as well. The dose threshold was set to 15% to ensure that only the target region was included in the comparison. Pass rates were compared for treatments for different field widths, rotation periods, and breathing patterns. Second, dose profiles were obtained from the individual diode detector readings along the length of the phantom. The dose profiles were examined for motion‐related artifacts, and the doses given to regions of interest, such as the OAR and the edges of the CTV, were calculated.

## III. RESULTS

The specific shape and level of the dose profiles for each plan as planned and measured by ArcCHECK in the static phantom were dependent on machine parameters and consequently varied slightly from the prescription dose. To eliminate the impact of these differences on evaluation of the effect of motion, the ‘coverage dose’ was defined for each individual plan as the dose value on a profile actually covering 95% of the CTV in superior–inferior direction in the static phantom measurements. The coverage doses varied from 3.6 Gy in 2.5/60 to 4.0 Gy in 1.0/60 plans. For different plans, the cold areas (5% of the target volume) appeared in different locations and for the 2.5/60 case, the cold spot was at the superior–inferior borders. This plan is still clinically acceptable as it fulfills the requirement of the prescribed dose to 95% of the volume. Since we wanted to investigate the impact of the target motion on dose coverage, it was convenient to introduce the notion of the coverage dose in order to concentrate on the effect of target motion in clinically relevant situations. The delivered plans were planned with final dose calculation that takes into account the limitation of minimum opening time for the MLC leaves; this is why our planned dose distributions were the same as measured with the static phantom. The maximum dose was generally below 110% of the coverage doses, with only 1.0/16 going above, at 114%. Most plans had peak doses on either edge of the CTV. Plans 2.5/60, 5.0/16, and 5.0/60 had maximum doses in the center for the CTV. Because the OAR was set on the ‘gantry’ side of the tumor, the dose levels tended to dip lower on this side of the CTV. Reciprocally, the dose levels in the OAR were always at a maximum on the ‘foot’ side of the OAR, next to the CTV.

**Figure 3:**
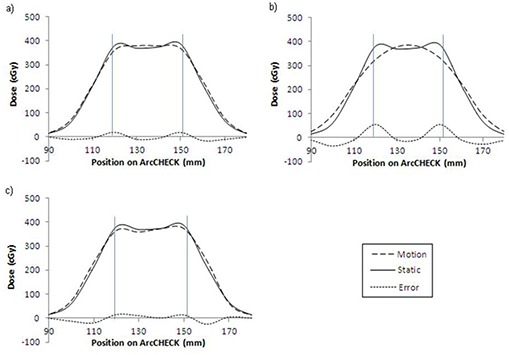
Solid lines = dose profiles for static measurements, dashed lines = dose profiles for motion measurements, dotted line = the difference between the static and motion measurements, vertical lines = the CTV boundaries. Dose distributions at the target for the 2.5 cm field width, 30 s rotation period plan with: a) 5 mm of sinusoidal motion, b) 10 mm of sinusoidal motion, and c) real patient breathing motion shown in Fig. 2.

Figure 3 shows dose profiles for the 2.5/30 plan, with the phantom moving with a) 5 mm amplitude sinusoidal, b) 10 mm amplitude sinusoidal, and c) real patient breathing waveform with average amplitude slightly less than 5 mm.

The dose distribution resulting from the 10 mm motion amplitude shown in Fig. 3(b) shows a much greater amount of rounding than in the case of 5 mm amplitude motion shown in Fig. 3(a). This trend of increased rounding with increasing tumor motion amplitude was true of all plans, with more significant effects for smaller field widths. Rounding was the only detrimental effect of sinusoidal motion within the CTV, causing cold spots to occur on either side of the CTV, but never in the center.

The results for treatments with real patient breathing tumor motion were somewhat similar. The dose distributions were still rounded, but with less uniformity and predictability, resulting in slightly different effects for each plan. However, even with this type of tumor motion, there were no significant dose discrepancies within the CTV.

There were some significant dose discrepancies outside of the CTV for plans involving sinusoidal tumor motion. Although the dose distribution retained the generally correct shape, it was displaced with respect to the intended distribution, resulting in shifts as large as the tumor motion amplitude to either side. The doses in these areas were quite low, so although the percent difference between the motion and static doses were very high, the absolute dose differences were small in comparison to differences on the edges of the CTV.

**Figure 4:**
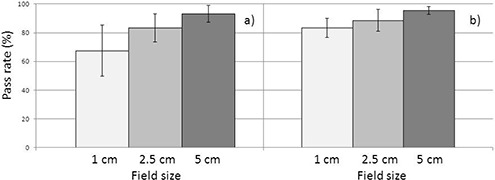
Average pass rate for plans with different field width for (a) sinusoidal and (b) real patient motion patterns. Error bars correspond to 1 SD.

The percentage of points passing the gamma criteria, averaged over both motion amplitudes and rotation periods for all measurements of plans of different field widths, is shown in Fig. 4 for sinusoidal motion breathing patterns (a) and real patient breathing patterns (b). The large size of standard deviations reflects the range of passing rates for cases with different motion amplitudes and gantry rotation periods. There is a positive correlation between increasing field width and increasing passing rate in both cases, with Spearman correlation ρ between 0.77 and 0.96 (p<0.02). A rotation period aligned Student two‐tailed paired t‐test for passing rates comparison resulted in statistically significant difference (p<0.001) between deliveries with 1 and 2.5 cm FWs for all rotation periods, and between deliveries with 2.5 and 5 cm FWs for rotation periods of 16 and 30 s.

**Figure 5:**
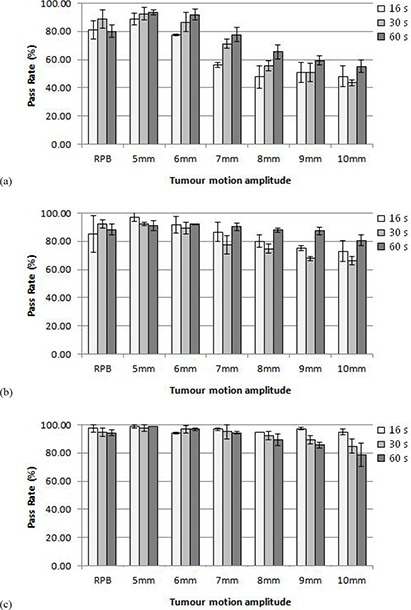
Average pass rate for plans with different gantry rotation periods and with a) 1 cm, b) 2.5 cm, and c) 5 cm field sizes for target sinusoidal motion with different amplitudes and with a real patient motion pattern (RPB). Error bars correspond to 1 SD.

The choice of the rotation period, however, has affects on delivered dose distribution with different field widths. Figures 5(a), (b), and (c) show the average passing rate per rotation period for each of the three field widths studied. At each field width, there is a specific pattern of passing rates vs. rotation period which is consistent over most amplitudes. However, the patterns are not apparent in the real patient breathing treatments. The overall trend caused by changing field widths is still seen at each individual rotation period.

**Figure 6:**
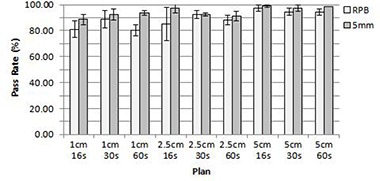
Comparison of pass rates averaged over three measurements for real patient breathing (RPB) and sinusodal with 5 mm amplitude motion patterns for different combinations of tomotherapy plan FW and RP parameters. Error bars correspond to 1 SD.

The treatments with real patient breathing tumor motion of 5 mm average amplitude had approximately the same passing rate as the 5 mm amplitude sinusoidal breathing pattern for every plan delivered three times, as shown in Fig. 6.

The dose prescription was set as 4 Gy to 95% of the CTV and when we measured dose distribution with motion, the profile was blurred at the sides, so that the distance between the points corresponding to 95% of the prescription dose in the static and “moving” cases was defined as a margin needed to add to the CTV in order to account for motion.

**Figure 7:**
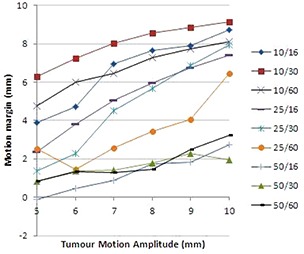
Estimated motion margin required for compensation of the rounding effect for the plans with different FW/RP parameters.

The obtained motion margins for each set of parameters are shown in Fig. 7. The required margin size increases with decreasing field width and increasing tumor motion amplitude, but is not affected by rotation period. The plans with the smallest field width of 1 cm have required motion margins approximately equal to the amplitude of motion (±25%), while those with the largest field width of 5 cm have required motion margins approximately equal to 20% of the motion amplitude (±20%).

For small tumor motion amplitudes and field widths above 1 cm, the required motion margins are very small — at a maximum of 2.5 mm for sinusoidal breathing patterns and 1.2 mm for the real patient breathing pattern.

**Figure 8:**
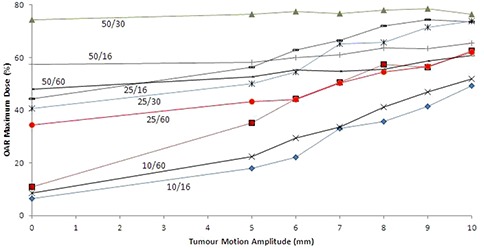
Organ‐at‐risk maximum doses (in percentage of the PSPD) as a function of tumor motion amplitude for different sets of tomotherapy FW/RP parameters.

Figure 8 shows the maximum dose received by the OAR for each set of machine parameters as a function of tumor motion amplitude once the proposed motion margins are added in. The plans with smaller field widths result in lesser doses to the OAR with small amounts of motion, but the difference between different sets of machine parameters decreases as the tumor motion amplitude increases.

## IV. DISCUSSION

The results of this study indicate that motion may result in a nonnegligible dosimetric impact for some, but not all, machine and tumor motion parameters. As expected, the greatest dose discrepancies occurred in treatments with small field widths and large tumor motion amplitudes. Differing rotation periods had no direct effect.

Kim et al.^(^ ^6^ ^)^ identified three types of artifacts which may be caused by motion in HT: dose rounding, dose rippling, and the leaf‐opening asynchronization effect. In the present study, for both sinusoidal and real breathing target motions, dose rounding was observed for every measurement; however, there was no dose rippling or leaf‐opening asynchronization effect. The lack of rippling can be explained by the fact that the ratios of the time required for the couch to travel a distance of FW to the target motion period used for this study ranged from 17.4 to 27.0, far above the value of 4.5 at which rippling becomes insignificant.^(^ ^6^ ^)^ The lack of asynchronization effect may be due to the fact that the same tumor motion period of 4 s was used for all sinusoidal treatments and 3 s for all real patient breathing treatments. All plans also had relatively simple sinograms, which included little variation in leaf‐opening. Additionally, the tumor size may have been too small for the effect to occur, or it may not have been detected due to the diode spacing of 1 cm. A more thorough investigation should be conducted using more breathing patterns and differing treatment plans to determine under what conditions this effect may be observed. Several other authors have found no significant dose difference using sinusoidal waveforms.^(^ ^9^ ^,^ ^10^ ^)^ Chaudhari et al.^(^ ^14^ ^)^ have observed more significant differences using real patient rather than sinusoidal breathing patterns between dose distributions for treatments to static versus moving targets. They have observed larger errors due to motion in real patients only for cases of the breathing pattern with a drift toward inhalation (motion drifting inferiorly). In our breathing pattern shown in Fig. 2, there is no such drift.

The significant dose discrepancies noted outside of the CTV for the sinusoidal waveforms can be explained. As the source rotates around the gantry, the beam always points towards the target. Thus, although the target remains in the beam for an extended period of time, the areas around the target are only irradiated for a moment as the beam passes through that angle. Should the detector (or tissue) be displaced during this moment of irradiation, the dose distribution will be offset. When the rotation period is a multiple of the tumor motion period, each pass of the source synchs with the others, and the actual dose distribution weaves back and forth across the intended distribution. With other rotation periods, different effects are seen. Thus, with thresholds lower than the 15% used in this study, comparisons of the low‐dose regions of motion versus static treatments would produce failed points, even with a fairly accurate CTV dose. This effect was not seen for the real patient breathing motion because the motion was not perfectly regular.

The margins required for plans with 1 cm beam width are shown to be approximately equal to the amplitude of tumor motion. For beam widths greater than 1 cm and motion with 5 mm amplitude or less, very small margins of only 2−3 mm may be acceptable. This was found for both sinusoidal and real patient breathing patterns. The margins for the real breathing patterns were actually slightly less than for the sinusoidal patterns because rounding was somewhat less significant. For moderately large motion amplitudes (5−10 mm), margins of 80% of the motion amplitude should suffice.

Others have suggested that the advantage of better sparing of sensitive tissues held by 1 cm field width plans may be negated by the increased margins.^(^ ^10^ ^)^ This is proven to be correct in our experiments. Chan et al.^(^ ^20^ ^)^ has shown that increased intensity at the edge of the target may reduce the effect of motion. The thread effect may have helped to reduce CTV underdosing in this study because of the hotspots it caused on the edges of the CTV.

## V. CONCLUSIONS

The impact of longitudinal motion on helical tomotherapy treatments was investigated using a 3D quality assurance phantom ArcCHECK on a moving platform. The passing rates for comparison between planned and measured doses were evaluated for periodic motion with amplitudes up to 10 mm and for a real patient breathing pattern. Motion margins for various machine parameters are recommended, and optimal machine parameters for treatments involving motion are suggested. Margins required to compensate for a blurring of the dose distribution to the target were found to be equal to the amplitude of motion for deliveries with 1 cm fan‐beam field size. For 2.5 and 5 cm field widths, a motion margin of 3 mm is sufficient for tumor motion less than 5 mm, and margin equal to 80% of the tumor motion amplitude is recommended for larger motion. Most effects are less pronounced for real patient breathing patterns compared to regular sinusoidal target motion.
